# Association between small vessel disease and slow gait speed in older adults with cognitive impairment

**DOI:** 10.1590/1980-5764-DN-2025-0313

**Published:** 2025-10-24

**Authors:** Mauricio Vazquez-Guajardo, Alberto José Mimenza-Alvarado, Luis Enrique Martínez-Bravo, Johnatan Rubalcava-Ortega, Manuel Montero-Odasso, Sara Gloria Aguilar-Navarro

**Affiliations:** 1 Universidad Nacional Autónoma de México, Facultad de Medicina, Department of Geriatrics, Instituto Nacional de Ciencias Médicas y Nutrición Salvador Zubirán, Mexico City, Mexico.; 2 Universidad Nacional Autónoma de México, Facultad de Medicina, Department of Neuroradiology, Instituto Nacional de Ciencias Médicas y Nutrición Salvador Zubirán, Mexico City, Mexico.; 3 Western University, Schulich School of Medicine & Dentistry, Division of Geriatric Medicine, Department of Medicine, London, ON, Canada.

**Keywords:** Cerebral Small Vessel Disease, Gait Disorders, Neurologic, White Matter, Stroke, Lacunar, Cognitive Dysfunction, Magnetic Resonance Imaging, Enfermedades de los Pequeños Vasos Cerebrales, Trastornos Neurológicos de la Marcha, Sustancia Blanca, Accidente Vascular Cerebral Lacunar, Disfunción Cognitiva, Imagen por Resonancia Magnética

## Abstract

Subjective cognitive decline and mild cognitive impairment are often associated with gait disturbances, increasing dementia risk. Cerebral small vessel disease (CSVD) may underlie these associations.

**Objective:**

To examine the association between CSVD lesion types and locations with slow gait speed in older adults with subjective cognitive decline and mild cognitive impairment.

**Methods:**

In this cross-sectional study, 124 older adults met the inclusion criteria. Participants underwent clinical evaluation, gait speed assessment, and brain magnetic resonance imaging. CSVD burden was assessed using the STRIVE-2 criteria and quantified using Fazekas and modified Scheltens scales. Logistic regression analyses were conducted to calculate odds ratios (OR) with 95% confidence intervals.

**Results:**

Individuals with slow gait were older, had lower education levels, and a higher prevalence of hypertension. Neuroimaging analysis revealed a significant association between slow gait and global white matter hyperintensities (WMH) burden (Fazekas score ≥2: 34.5 vs. 12.1%, p<0.003; Scheltens score ≥5: 65.8 vs. 42.4%, p<0.010). Regional WMH analysis showed increased burden in frontal and occipital regions in the slow gait group. Lacunar infarcts were more prevalent in the slow gait group (15.2 vs. 3.4%, p=0.028). Multivariate analysis revealed that lacunar infarcts and WMH in specific brain regions remained significant predictors of slow gait, even after adjusting for confounders.

**Conclusion:**

CSVD, particularly lacunar infarcts and specific WMH patterns, is associated with slow gait in this population. Early identification and management of CSVD may help mitigate its impact on gait and functional status.

## RESUMEN.

La queja subjetiva de memoria (QSM) y el deterioro cognitivo leve (DCL) suelen coexistir con alteraciones de la marcha, aumentando el riesgo de demencia. La enfermedad de pequeño vaso (EPV) podría explicar esta asociación.

**Objetivo:**

Examinar la relación entre los tipos y localizaciones de lesiones de EPV y la velocidad de marcha reducida en adultos mayores con QSM y DCL.

**Métodos:**

Estudio transversal en 124 adultos mayores que cumplieron criterios de inclusión. Se realizaron evaluaciones clínicas, medición de la velocidad de marcha y resonancia magnética cerebral. La carga de EPV se cuantificó con las escalas de Fazekas y Scheltens modificada. Se aplicaron análisis de regresión logística para calcular razones de momios (OR) con intervalos de confianza del 95%.

**Resultados:**

Los individuos con andar lento eran mayores, tenían menor nivel educativo y mayor prevalencia de hipertensión. El abdar lento se asoció con mayor carga de hiperintensidades de la sustancia blanca (HSB) (Fazekas ≥2: 34.5% vs. 12.1%, p<0.003; Scheltens ≥5: 65.8% vs. 42.4%, p<0.010) y con infartos lacunares (15.2% vs. 3.4%, p=0.028). El análisis regional mostró mayor carga de HSB en regiones frontales y occipitales en el grupo con marcha lenta. El análisis multivariado confirmó estas asociaciones tras ajustar por factores de confusión.

**Conclusión:**

La EPV, en especial los infartos lacunares y ciertos patrones de HSB, se asocia con la marcha lenta. Su detección y manejo temprano podrían mitigar su impacto en la función motora.

## INTRODUCTION

 Subjective cognitive decline (SCD) and mild cognitive impairment (MCI) often coexist with gait disturbances in older adults, contributing significantly to morbidity and mortality^1^. This combination, recently termed "motoric cognitive risk syndrome (MCR)" or "dual decliners," is associated with an elevated risk of dementia progression^2,3^. Cerebral small vessel disease (CSVD) is considered a potential underlying mechanism for these cognitive and motor deficits, disrupting subcortical motor networks essential for gait and balance^1^. 

 Gait disturbance is a prominent symptom of CSVD, often preceding cognitive decline^4^. CSVD affects small vessels in the brain, with diameters ranging between 50 and 500 μm^5,6^. Early identification of CSVD, up to a decade before dementia onset, offers a critical window for preventive interventions, especially in low- and middle-income countries^7, 8^. It affects neuronal circuits in subcortical regions that impact a wide range of organ systems. However, characterizing and assessing its burden in neuroimaging remains challenging due to the broad spectrum of lesions encompassed by the condition. International efforts now aim to better define these lesions through standardized criteria. These criteria include quantitative and qualitative evaluations of lesion morphology, extent, and location^9^. Among these, two lesions stand out in the context of cognitive decline and gait disorders: white matter hyperintensities (WMH) and lacunar infarcts^10-12^. The severity of WMH has been strongly associated with slower gait speeds, reduced balance performance, and higher odds of physical disability, with severe WMH burden correlating with pronounced gait and motor disturbances^13^. Similarly, lacunar infarcts, especially those in the frontal lobe and thalamus, independently contribute to reduced gait velocity and shorter stride length^10^. 

 This study aimed to investigate the association between CSVD lesion types and locations, as defined by the STRIVE-2 criteria^9^, and gait speed in older adults with SCD and MCI. We hypothesized that specific CSVD lesion patterns, particularly those affecting subcortical motor networks, are associated with slower gait speed. 

## METHODS

 This cross-sectional study included individuals aged 60 years or older with a diagnosis of MCI or SCD. MCI was diagnosed according to modified Petersen’s criteria^14-16^, and SCD was defined by the SCD Initiative (SCD2014) criteria^17,18^. Participants were recruited from a memory clinic between March 2023 and October 2024. All participants underwent clinical evaluation, gait speed assessment, and brain magnetic resonance imaging (MRI). 

 Participants were excluded if they had dementia, a history of stroke (ischemic or hemorrhagic), severe functional dependence (Katz Index score <5), musculoskeletal disorders limiting mobility (severe osteoarthritis, vertebral fractures due to osteoporosis, immobility syndrome, or a history of hip fracture), poorly controlled hypothyroidism (TSH >6), normal-pressure hydrocephalus, advanced heart failure (NYHA ≥3), severe chronic obstructive pulmonary disease — COPD (mMRC ≥3), Child B hepatic disease, or Kidney Disease: Improving Global Outcomes (KDIGO) IV nephropathy. 

 Clinical data were collected during routine visits. Cognitive function was assessed using the Mini-Mental State Examination (MMSE)^19^ and Montreal Cognitive Assessment (MoCA)^20^. Functional evaluations were conducted using the Katz Index (basic activities of daily living) and the Lawton & Brody Index (instrumental activities of daily living)^21,22^ . 

 The study population was divided into two groups based on gait speed measurements. Gait speed was measured following recommendations from the Mexican National Institute of Geriatrics and analyzed using the cutoff of <0.8 m/s for slow gait established by the European Working Group on Sarcopenia 2 (EWGSOP-2), the Asian Working Group on Sarcopenia (AWGS), and the Foundation of National Institutes of Health (FNIH)^23-26^. The definition provided by Fried’s frailty phenotype (<20% gait speed by height and gender) was not used to ensure an operational and practical definition suitable for daily use^27^. 

 Participants walked a standardized 4-meter distance marked on the floor, with two measurements taken during the visit. The fastest time was recorded in the clinical notes^23- 26^. Gait speed closest to the cranial MRI date was included in the analysis. 

 Images were obtained using a 1.5 Tesla General Electric (GE) SIGNA EXCITE scanner with axial fluid-attenuated inversion recovery (FLAIR), axial T2-weighted, volumetric T1-weighted, and susceptibility-weighted imaging (SWI) sequences. The FLAIR sequence had the following parameters: repetition time (TR) of 6,000 ms, echo time (TE) of 146.7 ms, inversion time (TI) of 1812 ms, acquisition matrix of 512 × 512, 30 axial slices, and a slice thickness of 5 mm. The SWI sequence included 88 axial slices, an acquisition matrix of 512 × 512, and a slice thickness of 3 mm. 

 CSVD burden was evaluated by two experienced neuroimaging researchers blinded to clinical information. Any discrepancies were resolved by a third expert. 

 CSVD burden was assessed using STRIVE-2 operational definitions^9^. The extent and location of white matter hyperintensities (WMH) were measured in axial FLAIR sequences using Fazekas’ visual scale (1: mild, 2: moderate, 3: severe)^28^ and Scheltens’ semiquantitative scale (0–84), which scores 0–6 across 13 subcortical regions (subcortical white matter, basal ganglia, infratentorial regions) and 0–2 in three periventricular regions^29^ (Supplementary Table S1). Lacunar infarcts were differentiated from perivascular spaces (PVS) and microbleeds using FLAIR, echo-gradient, T2, and susceptibility-weighted sequences. PVS have similar intensity to lacunar infarcts but differ in size (3–15 mm for lacunar infarcts vs. ≤2 mm for PVS) and morphology (lacunar infarcts are ovoid/spherical, while PVS follow blood vessels)^9^. Lacunar infarcts were categorized by region (frontal, parieto-occipital, temporal, basal ganglia, infratentorial)^30^, and PVS were graded semi-quantitatively (grade 1: 1–10; grade 2: 11–20; grade 3: 21–40; grade 4: >40)^8^. 

 Descriptive statistics were used to summarize participants’ characteristics. Continuous variables were presented as mean±standard deviation or median with interquartile range, depending on their distribution. Normality was assessed using the Kolmogorov-Smirnov test to determine whether parametric or non-parametric statistical tests were appropriate. Categorical variables were reported as frequencies and percentages. Comparative analysis between slow and normal gait groups used Pearson’s chi-square or Fisher’s exact test for categorical variables. Continuous variables were compared using Student’s t-test for parametric data or the Mann-Whitney test for non-parametric data. Hypertension was analyzed as a categorical variable (present/absent). To simplify the analysis of neuroimaging variables, Fazekas and Scheltens scales were grouped: Fazekas scores 0 and 1 were combined as "≤1," while scores 2 and 3 were grouped as "≥2." Similarly, Scheltens WMH scores were consolidated into two groups: <5 and ≥5. WMH in the 13 subcortical regions of Scheltens’ scale were classified as "0–3" or "4–6." Finally, lacunar infarcts were categorized as "lacunar infarcts (any site)". This grouping facilitated robust analysis by reducing variability and enabling clearer group comparisons. 

 Univariate and multivariate logistic regression analyses were conducted to calculate odds ratios (OR) with 95% confidence intervals. The variables chosen for the multivariable regression analysis were selected based on their statistical significance in preliminary analyses and their clinical relevance. All analyses were performed using the Statistical Package for the Social Sciences — SPSS^®^ software (version 25 for MacOS, Armonk, NY; IBM Corp.). This study was conducted in adherence to the Strengthening the Reporting of Observational Studies in Epidemiology (STROBE) guidelines^31^. 

 The protocol adhered to the current version of the Declaration of Helsinki and local regulatory requirements^32^. It was approved by the Ethics and Research Committees under the identification GER-5091-24-24134 and no financial or material support influenced the study design, data collection, analysis, or interpretation of results. 

## RESULTS

**Figure 1:**
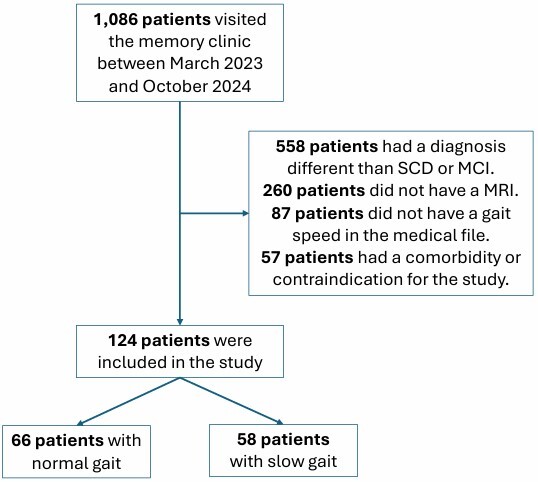
Participant selection flowchart. Abbreviations: SCD, subjective cognitive decline; MCI, mild cognitive impairment; MRI, magnetic resonance imaging.

 A total of 1,086 candidates were assessed at the memory clinic, of whom 962 were excluded for not meeting the inclusion criteria: 558 had a diagnosis other than SCD or MCI, 260 lacked a brain MRI, 87 had no gait speed data, and 57 had comorbidities or contraindications. The final sample included 124 participants, with 66 (53%) classified as having normal gait and 58 (47%) as having slow gait (Figure 1). The demographic characteristics of the participants are summarized below (Table 1). The mean age of the total cohort was 75 (± 7) years, with the slow gait group being significantly older (77 vs. 73 years, p<0.001). The distribution of female participants was 68.5% overall, with no significant difference between the gait groups (p=0.122). Height and body mass index (BMI) were similar across groups. Education levels were lower in the slow gait group, with a median of ten years compared to 12 years in the normal gait group (p=0.020). 

**Table 1:** Categorical variables are expressed as number and percentage (%), while continuous variables are presented as mean±standard deviation or median and interquartile range.

		Total (n=124)	Normal gait ≥0.8 m/s (n=66)	Slow gait <0.8 m/s (n=58)	p-value
Demographic characteristics					
	Age (years±SD)	75±7	73±6	77±7	0.001
	Female (n, %)	85 (68.5)	41 (62.1)	44 (75.6)	0.122
	Height (meters)	1.59±0.09	1.60±0.09	1.57±0.09	0.074
	BMI (kg/m^2^)	26.4±3.8	26.2±3.9	26.7±3.8	0.553
	Education (years, IQR)	12 (8–16)	12 (9–16)	10 (5–15)	0.020
Cognitive evaluation					
	Subjective cognitive decline (n, %)	32 (25.8)	18 (27.3)	14 (24.1)	0.691
	Mild cognitive impairment (n, %)	92 (74.2)	47 (71.2)	45 (77.6)	0.418
	MMSE (score, IQR)	27 (25–28)	27 (25–28)	27 (25–28)	0.785
	MoCA (score, IQR)	23 (18–25)	23 (20–25)	21 (17–25)	0.106
	Visuoespatial/executive functions (score, ±SD)	2.96±1.10	3.10±1.12	2.78±1.07	0.225
	Naming (score, ±SD)	2.66±0.55	2.74±0.44	2.56±0.66	0.165
	Atention (score, ±SD)	4.16±1.60	4.38±1.68	3.77±1.41	0.139
	Language (score, ±SD)	1.48±1.03	1.64±1.07	1.24±0.93	0.127
	Abstraction (score, ±SD)	1.21±0.91	1.44±0.88	0.91±0.88	0.011
	Delayed recall (score, ±SD)	1.91±1.61	2.14±1.58	1.61±1.62	0.155
	Memory index score (score, ±SD)	9.29±3.42	10.02±3.22	8.29±1.62	0.031
	Orientation (score, ±SD)	5.55±0.74	5.60±0.73	5.47±0.75	0.432
Gait speed		0.8 (0.6–0.9)	0.9 (0.9–1.1)	0.6 (0.5–0.7)	<0.001
Comorbidities (n, %)					
	Hypertension	64 (51.6)	27 (42.2)	37 (63.8)	0.017
	Diabetes mellitus	42 (33.9)	23 (34.8)	19 (33.3)	0.860
	Hyperlipidemia	46 (37.1)	24 (36.4)	22 (37.9)	0.857
	Obesity	26 (21.0)	13 (19.7)	13 (22.4)	0.711
	COPD	3 (2.4)	0 (0)	3 (5.2)	0.061
	Obstructive sleep apnea	11 (8.9)	7 (10.6)	4 (6.9)	0.469
	Depression	42 (33.1)	19 (28.8)	22 (37.9)	0.280
	Hypothyroidism	37 (29.8)	18 (27.3)	19 (32.8)	0.505
	Time from evaluation to neuroimaging (days, IQR)	233 (80–375)	244 (73–414)	207 (74–341)	0.982

Abbreviations: SD, standard deviation; BMI, body mass index; IQR, interquartile range; MMSE, Mini-Mental State Examination; MoCA, Montreal Cognitive Assessment; COPD, Chronic Obstructive Pulmonary Disease.

 The prevalence of MCI was similar in both groups (71.2% in normal gait vs. 77.6% in slow gait, p=0.418). MMSE scores showed no significant difference between groups, with median scores of 27 in both. MoCA scores were slightly lower in the slow gait group compared to the normal gait group, but this difference was not statistically significant (median 21 vs. 23; p=0.106). No significant differences were observed in executive functions. However, the slow gait group had significantly lower scores in abstraction (p=0.011) and memory index score (p=0.031). 

 Regarding comorbidities, hypertension was more prevalent in the slow gait group (63.8%) compared to the normal gait group (42.2%, p=0.017). There were no significant differences in other comorbidities between the groups. 

**Figure 2:**
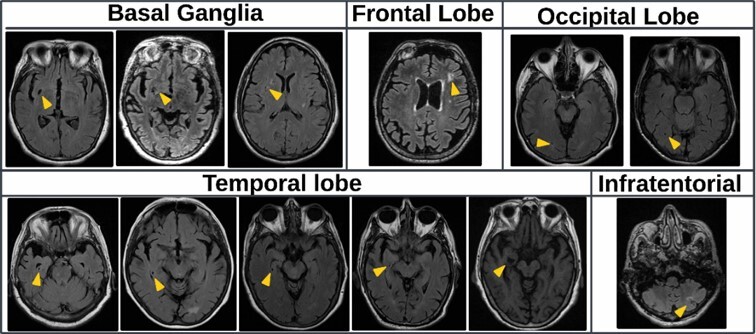
Localization and frequency of lacunar infarcts. This figure illustrates the 12 lacunar infarcts (arrowheads) identified in 11 of the 124 participants of the study, categorized by their localization in five predefined brain regions. Only one participant had 2 lacunar infarcts (temporal and basal ganglia). The frequency of lesions is represented by the number of images in each section, with 3 lacunar infarcts in the basal ganglia, 1 in the frontal lobe, 2 in the occipital lobe, 5 in the temporal lobe, and 1 in the infratentorial region. The images were obtained from fluid-attenuated inversion recovery (FLAIR) magnetic resonance imaging (MRI) sequences, highlighting the specific locations of the lacunar infarcts across these brain regions.

Neuroimaging findings (Table 2) showed that individuals with slow gait had more severe global WMH compared to those with normal gait as measured by the Fazekas scale ≥2 (34.5 vs. 12.1%; p < 0.003) and the Scheltens scale ≥5 (65.8 vs. 42.4%; p<0.010). Regional analyses from the Scheltens scale showed increased WMH in the frontal horns (37.9 vs. 15.2%; p<0.004), frontal lobes (27.6 vs. 9.1%; p<0.007), and occipital lobes (24.1 vs. 7.6%; p<0.011) in the slow gait group. Additionally, lacunar infarcts were more prevalent in the slow gait group (15.2 vs. 3.4%, p=0.028). Lacunar infarcts were found in the insula, basal ganglia, frontal lobe, occipital lobe and cerebellum (Figure 2). No significant differences were observed in the presence of microbleeds or perivascular spaces between the two groups.

**Table 2:** Categorical variables are expressed as number and percentage (%), while continuous variables are presented as mean±standard deviation or median and interquartile range.

Neuroimaging visual scales		Total (n=124)	Normal gait ≥0.8 m/s (n=66)	Slow gait <0.8 m/s (n=58)	p-value
Global cortical atrophy scale (score±SD)		1±1	1±1	2±1	0.336
Medial temporal atrophy scale (score±SD)		1±1	1±1	1±1	0.069
Koedam scale (score±SD)		1±1	1±1	1±1	0.218
Fazekas (n, %)					
	≤1	96 (77.4)	58 (87.9)	38 (65.5)	0.003
	≥2	28 (22.6)	8 (12.1)	20 (34.5)	
Scheltens WMH (n, %)					
	<5 pts	58 (46.8)	38 (57.6)	20 (34.6)	0.010
	≥5 pts	66 (53.2)	28 (42.4)	38 (65.8)	
WMH frontal horn (n, %)					
	≤1 pts	92 (74.2)	56 (84.8)	36 (62.1)	0.004
	2 pts	32 (25.8)	10 (15.2)	22 (37.9)	
WMH posterior horn (n, %)					
	≤1 pts	108 (87.1)	58 (87.9)	50 (86.2)	0.782
	2 pts	16 (12.9)	8 (12.1)	8 (13.8)	
WMH frontal lobe (n, %)					
	0–3 pts	102 (82.3)	60 (90.9)	42 (72.4)	0.007
	4–6 pts	22 (17.7)	6 (9.1)	16 (27.6)	
WMH parietal lobe (n, %)					
	0–3 pts	109 (87.9)	61 (92.4)	48 (82.8)	0.100
	4–6 pts	15 (12.1)	5 (7.6)	10 (17.2)	
WMH occipital lobe (n, %)					
	0–1 pts	105 (84.7)	61 (92.4)	44 (75.9)	0.011
	2–6 pts	19 (15.3)	5 (7.6)	14 (24.1)	
Lacunar infarct (any site)		12 (9.7)	2 (3.4)	10 (15.2)	0.028
Microbleeding (n=119)		9 (7.3)	3 (33.3)	6 (66.7)	0.201
Visible perivascular spaces centrum semiovale (n=103) (n, %)					
	0–2 pts	83 (66.9)	38 (45.8)	38 (45.8)	0.123
	3–4 pts	20 (16.1)	7 (35)	13 (65)	
Visible perivascular spaces basal ganglia (n=102) (n, %)					
	0–2 pts	83 (66.9)	43 (51.8)	40 (48.2)	0.445
	3–4 pts	19 (15.3)	8 (42.1)	11 (57.9)	

Abbreviations: SD, standard deviation; WMH, White Matter Hyperintensities; pts, points; WMH, White matter hyperintensities. Note: Statistical significance (p-values) indicates differences between the two groups.

 Logistic univariate regression analysis revealed that age ≥75 years, education (years), hypertension (present/absent), Fazekas score ≥2 points, Scheltens score ≥5 points, WMH in the anterior horn (2 points), WMH in the frontal lobe (4–6 points), WMH in the occipital lobe (2–6 points), and lacunar infarct (any site) were all significantly associated with slow gait (Table 3 ). 

**Table 3:** The table presents the univariate and multivariate analysis of factors associated with slow gait in older adults with subjective memory complaints and mild cognitive impairment. In the univariate analysis, odds ratios with 95% confidence intervals and p-values are shown for each variable. In the multivariate analysis, three models are presented, adjusted for different combinations of covariates: age ≥75 years, education, hypertension, lacunar infarct at any site, white matter hyperintensities anterior horn (2 points), Fazekas ≥2 points, and Scheltens ≥5 points, as appropriate.

Variable	Univariate		Multivariate					
			Model 1		Model 2		Model 3	
	OR (CI)	p-value	OR (CI)	p-value	OR (CI)	p-value	OR (CI)	p-value
Age ≥75 years	2.18 (1.05–4.53)	0.036	1.49 (0.66–3.39)	0.338	1.29(0.552.99)	0.555	1.37 (0.59–3.20)	0.462
Education (years)	1.07 (1.01–1.15)	0.033	1.05 (0.97–1.13)	0.205	1.04 (0.96–1.12)	0.314	1.05 (0.97–1.12)	0.462
Hypertension	2.41 (1.16–5.01)	0.018	1.92 (0.85–4.33)	0.115	2.36 (1.01–5.57)	0.049	1.87 (0.83–4.19)	0.129
Fazekas ≥2 pts	3.82 (1.53–9.54)	0.004			5.01 (1.26–19.94)	0.022		
Scheltens ≥5 pts	2.58 (1.24–5.34)	0.011					2.51 (1.09–5.77)	0.032
WMH anterior horn 2 pts	3.42 (1.45–8.06)	0.005	3.56 (1.32–9.58)	0.012	1.73 (0.54–5.53)	0.354		
WMH frontal lobe (4-6)	3.74 (1.35–10.37)	0.011						
WMH occipital lobe (2–6)	3.90 (1.30–11.66)	0.015						
Lacunar infarct (any site)	5.00 (1.05–23.86)	0.044	6.78 (1.25–36.99)	0.027	9.95 (1.62–61.21)	0.013	6.12 (1.17–32.02)	0.032

Abbreviations: OR, odds ratio; CI, confidence interval; p, probability value, pts: points; WMH, white matter hyperintensities. Notes: Model 1: Adjusted for age ≥75, education, hypertenlsion, lacunar infarct at any site, WMH anterior horn 2 points; Model 2: Adjusted for age ≥75, education, hypertension, lacunar infarct at any site, WMH anterior horn 2 points, Fazekas ≥2 pts.

 In the multivariate analysis, model 1 was adjusted for age, education, hypertension, WMH in the anterior horn, and lacunar infarcts. In this model, both WMH in the anterior horn (2 points) and lacunar infarcts were significant predictors of slow gait (OR 3.56; 95%CI 1.32–9.58; p=0.012 and OR 6.78; 95%CI 1.25–36.99; p=0.027, respectively). In model 2, which included additional adjustment for a Fazekas score ≥2, hypertension (OR 2.36; 95%CI 1.01–5.57; p=0.049), higher WMH burden on the Fazekas scale (≥2 points) (OR 5.01; 95%CI 1.26–19.94; p=0.022), and lacunar infarcts (OR 9.95; 95%CI 1.62–61.21; p=0.013) remained significantly associated with slow gait. Similarly, in model 3, which replaced the Fazekas score with the Scheltens scale, a higher WMH burden (≥5 points) (OR 2.51; 95%CI 1.09–5.77; p=0.032) and lacunar infarcts (OR 6.12; 95% CI 1.17–32.02; p = 0.032) continued to show a significant association with slow gait (Table 3). 

## DISCUSSION

**Figure 3:**
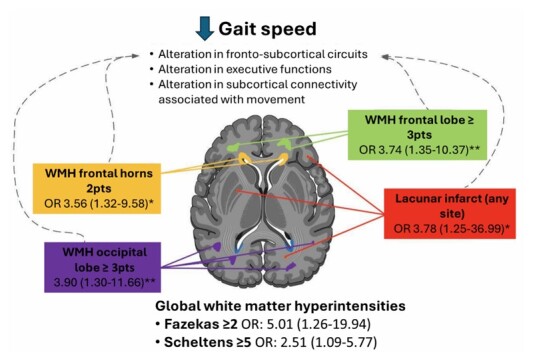
Findings of cerebrovascular lesions and their risk association with slow gait. The global burden of white matter hyperintensities (WMH) and their frontal predominance distribution were associated with slow gait. Lacunar infarcts at any site also showed an association with slow gait. Notes: ^*^Adjusted odds ratio (OR) for age, education, and hypertension; ^**^OR from univariate analysis.

 In this cross-sectional, retrospective study of 124 older adults with SCD and MCI, we identified a significant association between neuroimaging markers of global and regional CSVD and slow gait. Notably, it was observed that the global burden of white matter hyperintensities and lacunar infarcts at any site were associated with slow gait (Figure 3). 

 Gait is a complex process that involves the coordinated interaction of cortical functions, subcortical neural networks, the brainstem, spinal cord, and the locomotor system^33^. CSVD can affect this intricate balance by disrupting complex subcortical white matter circuits, as well as primary cortical motor and sensory areas, altering the final regulation of movement, gait fluency, movement planning and execution, ultimately resulting in reduced gait speed^12^. 

 Many risk factors influence cognition, gait speed and CSVD^34-37^. In this study, we applied careful exclusion criteria — particularly related to functional status (Katz Index <5) — to reduce potential confounding from functional dependence, which is closely linked to both dementia and mobility impairment. As expected, participants with slow gait were older, had lower education levels, and showed a higher prevalence of hypertension, consistent with existing literature^35^. Age is a critical risk factor for cognitive decline, slow gait speed and CSVD, with up to 96% of adults over 90 years showing evidence of small vessel lesions and a notable decline in gait speed, particularly after the age of 80^34-38^. A recent systematic review identified low education as one of the most significant risk factors for slow gait speed among other 85 studied factors. However, the specific causal mechanisms linking low educational attainment to increased risk of gait speed impairment remain poorly understood. It is theorized that individuals with fewer years of education are more likely to lead sedentary lifestyles, engage in unhealthy behaviors, and face barriers to healthcare, thereby increasing the likelihood of health declines and associated physical impairments, such as slow gait^35^. In addition to age and education, hypertension is a major risk factor for CSVD and has been shown to accelerate gait slowing in older adults^34 ,39^. 

 Even though these risk factors influence gait speed and CSVD, in the multivariable analysis adjusted to control for these confounding factors, the relationship between the global burden of WMH, anterior horn WMH, and lacunar infarcts with decreased gait speed remained. 

 Previous reports found that approximately 30% of patients with amnesic MCI have gait dysfunction. In our study, 43% of SCD patients and 49% of MCI patients exhibited slow gait speed, with no significant differences in cognitive impairment severity between the slow and normal gait groups. While previous research linked worsening gait parameters to cognitive decline in Alzheimer’s disease and vascular dementia, even at early MCI stages, we found no difference between SCD and MCI subgroups^4^. This might be explained by the mild nature of cognitive impairment in both groups, as more pronounced deficits, such as those in dementia, likely show stronger associations with gait speed^40,41^. The lack of differences could also stem from the cognitive assessment tools used, that failed to detect subtle variations in different cognitive domains that could impact gait. Future research encompassing a broader cognitive spectrum and employing more sensitive neuropsychological tools may uncover nuanced relationships between cognitive status and gait^42,43^ . 

 In this study we found that both global burden of WMH and lacunar infarcts were independently associated with slow gait speed. Beyond the known association of WMH with falls, our findings indicate that frontal horn and deep frontal WMH are linked to slow gait, likely due to disruption of long loop reflexes critical for gait and balance^13^. Other frontal lesions, such as subdural hematomas, tumors, or hydrocephalus, are well known to cause gait and balance disturbances, further supporting the idea that WMH in these regions could similarly affect frontal cortico-subcortical circuits responsible for motor control^44^. Additionally, lacunar infarcts in various regions, including the insular cortex and basal ganglia, showed a robust independent association with slow gait, highlighting their clinical relevance despite often being detected incidentally. These "covert infarcts" are more common than clinical strokes and can significantly impact cognitive and motor functions, underscoring the need for intentional investigation of these lesions^45,46^. The insular cortex processes a wide range of sensory signals arising from the body and integrates them with the emotional and motivational context. In doing so, it provides the impetus to the dorsomedial frontal cortex to initiate and sustain movement^47^. Lesions in the insular cortex have been associated with worse time-up-and-go (TUG) performance in stroke patients^48,49^. Meanwhile, basal ganglia involvement in gait and movement regulation has been extensively documented^10,50^. 

 Our study presents several limitations, and the results should be interpreted with caution. Its cross-sectional design restricts the ability to establish causal relationships, limiting the study to identifying associations. Although in our univariate analysis several covariates — such as diabetes, dyslipidemia and other comorbidities — were not statistically significant and therefore were not included in the multivariate model, we recognize that residual confounding cannot be fully ruled out. These factors may still influence both gait speed and CSVD burden through complex mechanisms. Additionally, the evaluation of gait speed and MRI imaging were not performed simultaneously, introducing a potential temporal bias. The analysis of CSVD was based solely on visual scales, excluding more sophisticated methods such as automated volumetric analysis, quantitative susceptibility mapping (QSM), diffusion-weighted imaging (DWI), or cerebral perfusion measures. The lack of longitudinal follow-up also prevents determining the relationship between MRI findings and clinical outcomes such as progression to dementia or functional decline. 

 Despite these limitations, the study has notable strengths. The use of MRI and validated visual scales like Fazekas and Scheltens allows for an objective and comparative assessment of CSVD. Furthermore, the inclusion of a clinically relevant population at risk of progressing to dementia, along with multivariate analyses to control for important confounders such as age, hypertension, and education, enhances the study’s robustness. Finally, the study provides novel findings on the association between specific regions of WMH and slow gait, offering a solid foundation for future longitudinal research. 

 In conclusion, this study highlights the significant association between cerebral small vessel disease (CSVD), particularly lacunar infarcts and regional white matter hyperintensities (WMH), and slow gait in older adults with SCD and MCI. The findings underscore the clinical relevance of these seemingly minor lesions, often detected incidentally, and their potential impact on gait and overall functional status. While the study’s retrospective and cross-sectional design limits causal inferences, the use of validated MRI scales and multivariate analyses provides robust evidence for the independent association of CSVD with slow gait. Future longitudinal studies are warranted to further elucidate the mechanisms underlying these associations and to explore potential therapeutic interventions aimed at mitigating the impact of CSVD on gait and cognitive function in older adults. 

## Data Availability

The datasets generated and/or analyzed during the current study are available from the corresponding author upon reasonable request.
