# Supplementary material for: Association between small vessel disease and slow gait speed in older adults with cognitive impairment
Source: Dement Neuropsychol. 2025 Oct 24;19:e20250313. doi: 10.1590/1980-5764-DN-2025-0313 (PMC12561174; doi:10.1590/1980-5764-DN-2025-0313)
Supplement: Supplementary Table S1 [file 1980-5764-dn-19-e20250313-md1.docx]

# Supplementary Material

**Table S1.** Adaptation of the Scheltens visual scale for the evaluation of white matter and basal ganglia hyperintensities on T2 FLAIR-weighted images. Scores are assigned based on the size and number of lesions in each region.

| **Periventricular White Matter Hyperintensities (0-6 points)** | | |
| --- | --- | --- |
| Anterior Horn Lateral Ventricle | 0/1/2 | Operational definitions:   - 0: absent. - 1: ≤ 5 mm. - 2: > 5 mm. |
| Posterior Horn Lateral Ventricle | 0/1/2 |  |
| Ventricular lateral bands | 0/1/2 |  |
| **White Matter Hyperintensities (0-24 points)** | | |
| Frontal | 0/1/2/3/4/5/6 | - 0: no abnormalities - 1: <3 mm, n ≤ 5 lesions. - 2: <3 mm, n >6 lesions - 3: 4-10 mm, n ≤ 5 lesions - 4: 4 mm-10 mm, n > 6 lesions - 5: >11mm, n >1 lesion - 6: confluent |
| Parietal | 0/1/2/3/4/5/6 |  |
| Occipital | 0/1/2/3/4/5/6 |  |
| Temporal | 0/1/2/3/4/5/6 |  |
| **Basal Ganglia Hyperintensities (0-30 points)** | |  |
| Caudate nucleus | 0/1/2/3/4/5/6 |  |
| Putamen | 0/1/2/3/4/5/6 |  |
| Globus pallidus | 0/1/2/3/4/5/6 |  |
| Thalamus | 0/1/2/3/4/5/6 |  |
| Internal Capsule | 0/1/2/3/4/5/6 |  |
| **Infratentorial White Matter Hyperintensities (0-24 points)** | |  |
| Cerebellum | 0/1/2/3/4/5/6 |  |
| Mesencephalon | 0/1/2/3/4/5/6 |  |
| Pons | 0/1/2/3/4/5/6 |  |
| Medulla oblongata | 0/1/2/3/4/5/6 |  |
